# Unmet non-medical needs of cancer patients in Poland: a quantitative and qualitative study

**DOI:** 10.1007/s00520-024-08387-5

**Published:** 2024-02-22

**Authors:** Karolina Osowiecka, Marek Szwiec, Anna Dolińska, Anna Gwara, Marcin Kurowicki, Jarosław Kołb-Sielecki, Eliza Działach, Weronika Radecka, Sergiusz Nawrocki, Monika Rucińska

**Affiliations:** 1 Department of Psychology and Sociology of Health and Public Health, School of Public Health, University of Warmia and Mazury in Olsztyn, Warszawska 30, 10-082 Olsztyn, Poland; 2 Department of Surgery and Oncology, Faculty of Medicine and Health Sciences, University of Zielona Gora, Zyty 28, 65-046 Zielona Gora, Poland; 3 Psychology Outpatient Clinic, University Hospital in Zielona Gora, Zyty 26, 65-046 Zielona Gora, Poland; 4 Department of Nursing, Institute of Health Science, University of Zielona Gora, Zyty 28, 65-046 Zielona Gora, Poland; 5 NU-MED Radiotherapy Center in Elblag, Królewiecka 146, 82-300 Elblag, Poland; 6 Department of Oncology, The Center for Pulmonary Diseases in Olsztyn, Jagiellońska 78, 10-357 Olsztyn, Poland; 7 Departament of Public Health, Faculty of Sciences in Bytom, Medical University of Silesia in Katowice, Piekarska 18, 41-902 Bytom, Poland; 8 Department of Anatomy, University of Opole, Kopernika 11a, 45-040 Opole, Poland; 9 Department of Oncology, Collegium Medicum University of Warmia and Mazury in Olsztyn, Wojska Polskiego 37, 10-228 Olsztyn, Poland

**Keywords:** Cancer, NEQ, Non-medical needs, Unmet needs, Information

## Abstract

**Purpose:**

Cancer itself and its treatment have a multifaceted impact on patients’ daily lives. The aim of the study was to determine unmet non-medical needs among Polish cancer patients.

**Methods:**

Survey research using a 23-item Needs Evaluation Questionnaire (NEQ) was carried out among 1062 cancer patients from different regions of Poland. Quantitative and qualitative analyses were performed.

**Results:**

The quantitative analysis showed that 48% of the NEQ items (11/23) were expressed as unmet needs by at least half of patients. Unmet information needs were indicated by patients most often: information about their diagnosis, exams, treatment, future condition, funding and economic support. Cancer patients would like to get more attention from medical staff. Unmet needs were most frequently expressed by respondents who were men, with a lower level of education, living in village, pensioners. Qualitative analysis showed that each need may be understood in a variety of different ways across the cohort. Some patients added comments that the completing NEQ helped them to notice their non-medical needs.

**Conclusion:**

Polish cancer patients have some unmet non-medical needs, especially informative needs.

**Supplementary Information:**

The online version contains supplementary material available at 10.1007/s00520-024-08387-5.

## Introduction

Cancer diagnosis is a new and stressful situation for everyone and affects all aspects of life. Patients with a cancer diagnosis face multiple problems, not only medical, but also logistical, social, psychological, and spiritual. Patients have a number of questions, concerns and are uncertain about the future [1, 2]. Cancer treatment consists of variety of therapies (surgery, radiotherapy, chemotherapy, immunotherapy, biological therapy, hormonal therapy), takes a lot of time (stay at hospital after operation, multifractionated radiotherapy, systemic treatment administered in intervals), is received in a place often unfamiliar to the patient (oncological departments), and often requires frequent visits to the out-patients clinic, and potentially multiple hospitalizations. Moreover, oncological therapy is often associated with numerous stigmatizing side effects (for example hair loss, cachexia, amputations) [3]. Patients can feel tired and overwhelmed by the disease and its treatment. Cancer usually leads to changes in patients' daily lives [4]. They may be unable to adequately fulfill their usual roles and functions within family, social and professional life. This can lead to changes in their needs and priorities and for new ones to emerge. Therefore, a holistic approach to oncology health care should be provided. Needs other than those directly related to diagnosis and treatment (social, material, informative, emotional, spiritual, ect.) often seem to be unmet [5]. The physicians and nurses often do not have enough time and opportunity to respond to the patient's non-medical needs. On the other hand, not all patients are able to express their non-medical needs [6]. Non-medical needs may be defined as those that do not directly relate to the disease and treatment and have no clinical or other medical relevance. It is a challenge to introduce the routine assessment of unmet needs of all cancer patients into daily clinical practice. An appropriate tool is needed to help patients better identify their needs and medical staff to gain a better understanding of the non-medical needs of cancer patients. Better understanding of cancer patients’ needs could facilitate the patient-doctor relationship and could improve both patients’ quality of life and their satisfaction with health care [7–9]. Therefore, more attention should be paid to patients' non-medical needs.

It seems to be crucial to introduce an appropriate, simple and easy to use in daily practice tool to assess cancer patients’ unmet needs. There are however some existing instruments to assess cancer patients’ needs [7].

Wen and Gustafson [7] have done detail analysis of existing questionnaires. Among tools analyzed by them was the Needs Evaluation Questionnaire (NEQ). The NEQ seems to be an appropriate, simple, comprehensive and easy to administer tool. The NEQ was created and validated by Tamburini et al. [10, 11] among Italian cancer patients. The NEQ has also been used in some other studies [12–15]. In Poland, the NEQ was used only once to assess the needs of hospice patients and their relatives [16].

The NEQ is a self-administered questionnaire and includes questions related to five areas of needs: informative needs, psycho-emotional needs, relational needs, material needs and needs related to assistance/care [17].

The aim of the study was to determine Polish cancer patients’ unmet non-medical needs using the NEQ.

## Materials and methods

### Participants

The study was carried out on a group of 1062 cancer patients treated in the period between June 2022 to May 2023 across seven oncological centers in Poland (University Hospital in Zielona Gora, *n* = 296; Hospital of the Ministry of Internal Affairs with Warmia and Mazury Oncology Center in Olsztyn, *n* = 85; The Center for Pulmonary Diseases in Olsztyn, *n* = 74; Hospital in Prabuty, *n* = 47; NU-MED Radiotherapy Center in Elblag, *n* = 424; Oncology Center in Opole, *n* = 66; Zaglebiowskie Oncology Center in Dabrowa Gornicza, *n* = 70).

The inclusion criteria were: aged ≥ 18 years old, pathologically confirmed cancer diagnosis, oncology treatment ongoing or patient having finished treatment no longer than 3 months previously, current hospitalization for at least 3 days or at least one hospitalization due to oncological treatment within the previous 3 months. The criterion of current or past hospitalization was included so that participants have potentially experienced problems related to hospitalization. The exclusion criteria were: age < 18 years old, no pathological confirmation of cancer, patients having finished treatment longer than 3 months previously, no hospitalization due to oncological treatment within the previous 3 months.

The participation in the study was proposed to the patients in the out- and inpatient oncological departments personally by psychologists or nurses. The survey was carried out using paper form of questionnaire. A part of participants completed the questionnaire themselves. However some patients needed support from medical staff (individuals in worse general condition, problems with vision, problems with writing, preference for talking).

### Questionnaire

The study was conducted using the Needs Evaluation Questionnaire (NEQ). Permission for use of the NEQ to assess unmet needs of Polish cancer patients included in this study was obtained from the authors of original version of the NEQ [10, 11]. The Polish version of the NEQ was developed with a back-translation procedure. The psychometric properties of the Polish version of questionnaire were previously evaluated on a group of 121 cancer patients (K.O., A.D., M.S., E.D., J.N., M.R., unpublished). The pilot study to determine unmet non-medical needs was conducted among Polish male lung cancer patients [18].

The NEQ consists of 23 items with responses on a dichotomous scale (yes/no). The questionnaire was supplemented by 10 questions concerning demographic data (age, gender, education, place of residence, professional activity, marital status, living alone or with someone, having a doctor amongst family/friends) and clinical data (type of cancer and approximate date of cancer diagnosis).

Polish and English versions of the NEQ are presented in Supplementary Materials.

### Quantitative analysis

The analysis was performed on a group of 1180 cancer patients, who met the inclusion criteria and decided to take part in the study. The response rate was 90%—1062 patients completed the NEQ. The percentages of answers “yes” and “no” for all individual items were calculated.

### Qualitative analysis

Of the 1062 patients who completed the questionnaire by themselves, 71 patients from two centers (University Hospital in Zielona Gora and Zaglebiowskie Oncology Center in Dabrowa Gornicza) were randomly selected for semi-structured interview with a psychologist.

#### Understanding of the meaning of the unmet non-medical needs

Items which were expressed as unmet needs by half or more participants were included to qualitative analysis.

There were eleven such items: Q1-4, Q6, Q7, Q9, Q13-15, Q21. These questions were used for assessing patients' understanding of the items and determining exactly what they meant by answering "yes". Additional questions about these 11 items were asked by a psychologist during a semi-structured interview.

#### Evaluation of the questionnaire

Subjective evaluation of the questionnaire based on patients' opinions was made by a psychologist during interview.

The comprehensibility and acceptability of the NEQ for patients were evaluated using a validation procedure questionnaire (VPQ) designed for the study. Polish and English versions of the VPQ are presented in Supplementary Materials.

The study protocol was approved by the Ethics Committee of the University of Warmia and Mazury in Olsztyn (No. 30/2020). Participation in the study was voluntary. All study participants were informed about the aim of the study and gave their consent and signed it.

### Statistical analysis

Descriptive statistics were performed. The distribution of cancer patients’ unmet non-medical needs was determined. The associations between unmet needs and demographics were determined using the non-parametric Mann–Whitney test for continuous variable and the chi-square test for categorical variables. The normal distribution of continuous variable was tested using the Shapiro–Wilk test. A p-value of < 0.05 was considered to be significant. The odds ratio (OR) with 95% confidence interval (CI) for expressing non-medical needs (Q1-23) according to demographic factors was estimated using logistic regression models. The variables with *p* ≤ 0.1 in univariate analysis were included to multivariate model. The data analysis was conducted using Statistica (data analysis software), version 13. http://statistica.io TIBCO Software Inc., Krakow, Poland (2017).

## Results

### Characteristics of patients

The study was conducted on a group of 1062 cancer patients with ages between 22 and 89 years old (median age 66 years). Patients were 48% women and 52% men. All participants were diagnosed with cancer. The most frequent cancers were lung cancer (28.2%), lower digestive system cancers (19.5%) and breast cancer (15.6%). Patients were 6 months from cancer diagnosis (median). All patients were under oncological treatment at the time of the study or finished treatment no longer than 3 months previously. The majority of patients had graduated from secondary school (68.1%), lived in cities (63.6%), were pensioners (73.4%), married (67.8%), living with partner (66.5%). Only 15.1% of participants had a physician in their close family or among friends (Table 1).

**Table 1:** Study group demographics

	*n*	%
Gender		
female	507	47.7
male	553	52.1
no data	2	0.2
Age: range 22–89 years, median 66 (59–71) years (25–75% IQR)		
Education		
primary	180	17.0
secondary	723	68.1
high	147	13.8
no data	12	1.1
Place of residence		
city	676	63.6
village	383	36.1
no data	3	0.3
Professional activity		
active	236	22.2
unemployed	41	3.9
pensioner	780	73.4
no data	5	0.5
Marital status		
married/in a stable relationship	720	67.8
relationship broken down during disease or in relation to disease	5	0.5
single	332	31.2
no data	5	0.5
Living with		
partner	706	66.5
child/children and/or another family member	166	15.6
alone	171	16.1
no data	19	1.8
Physician in close family or friends		
yes	160	15.1
no	893	84.1
no data	9	0.8
Cancer		
head and neck	62	5.9
upper digestive system	71	6.7
lower digestive system	207	19.5
lung	300	28.2
breast	166	15.6
gynecological	55	5.2
prostate	109	10.3
brain	15	1.4
urinary system	30	2.8
other	31	2.9
no data	16	1.5
Time from cancer diagnosis: range 1–265 months, median 6 (3–13) months (25–75% IQR)		

*IQR* interquartile range

### Needs prevalence – quantitative analysis

Eleven needs (48% of the 23 items assessed) were expressed as unmet by half or more cancer patients. Most of them were kind of informative needs (9/11 needs). The most frequently indicated needs related to information were: needs for more information about their future condition (68.7%; Q2) and their diagnosis (50%; Q1), need for more explanations about their treatments (59.9%; Q4) and the examinations they are undergoing (54.6%; Q3). Patients needed to be more reassured by clinicians (57.8%; Q13), had need for clinicians to be more sincere (57.3%; Q7) and for clinicians and nurses to give them more comprehensible information (52.3%; Q6). About half of respondents indicated the need for better control of their symptoms (51.3%; Q9) and for better services from the hospital (bathrooms, meals, cleaning) (55.6%; Q14. Most of participants expressed the need to have more information about economic insurance in relation to their illness (60.7%; Q15). Among relational needs about half of cancer patients (53.4%; Q21) indicated the need to feel more useful within their families (Table 2).

**Table 2:** The distribution of patients’ needs

Item number			n	%
Q1	I need more information about my diagnosis	yes	531	50.0
		no	525	49.4
		missing data	6	0.6
Q2	I need more information about my future condition	yes	729	68.7
		no	321	30.2
		missing data	12	1.1
Q3	I need more information about the exams I am undergoing	yes	580	54.6
		no	463	43.6
		missing data	19	1.8
Q4	I need more explanations of treatments	yes	636	59.9
		no	405	38.1
		missing data	21	2.0
Q5	I need to be more involved in the therapeutic choices	yes	496	46.7
		no	532	50.1
		missing data	34	3.2
Q6	I need clinicians and nurses to give me more comprehensible information	yes	555	52.3
		no	489	46.0
		missing data	18	1.7
Q7	I need clinicians to be more sincere with me	yes	608	57.3
		no	437	41.1
		missing data	17	1.6
Q8	I need to have a better dialogue with clinicians	yes	508	47.8
		no	535	50.4
		missing data	19	1.8
Q9	I need my symptoms (pain, nausea, insomnia, etc.) to be better controlled	yes	545	51.3
		no	500	47.1
		missing data	17	1.6
Q10	I need more help with eating, dressing, and going to the bathroom	yes	132	12.4
		no	914	86.1
		missing data	16	1.5
Q11	I need better respect for my intimacy	yes	296	27.9
		no	746	70.2
		missing data	20	1.9
Q12	I need better attention from nurses	yes	311	29.3
		no	728	68.5
		missing data	23	2.2
Q13	I need to be more reassured by the clinicians	yes	614	57.8
		no	422	39.7
		missing data	26	2.5
Q14	I need better services from the hospital (bathrooms, meals, cleaning)	yes	590	55.6
		no	438	41.2
		missing data	34	3.2
Q15	I need to have more economic insurance information (tickets, invalidity, etc.) in relation to my illness	yes	644	60.7
		no	390	36.7
		missing data	28	2.6
Q16	I need economic help	yes	330	31.1
		no	702	66.1
		missing data	30	2.8
Q17	I need to speak with a psychologist	yes	254	23.9
		no	774	72.9
		missing data	34	3.2
Q18	I need to speak with a spiritual advisor	yes	216	20.3
		no	813	76.6
		missing data	33	3.1
Q19	I need to speak with people who have this same experience	yes	426	40.1
		no	609	57.3
		missing data	27	2.6
Q20	I need to be more reassured by my relatives	yes	389	36.6
		no	640	60.3
		missing data	33	3.1
Q21	I need to feel more useful within my family	yes	567	53.4
		no	468	44.1
		missing data	27	2.5
Q22	I need to feel less abandoned	yes	364	34.3
		no	664	62.5
		missing data	34	3.2
Q23	I need to receive less commiseration from other people	yes	377	35.5
		no	663	62.4
		missing data	22	2.1

### Needs prevalence by demographic factors

#### Age

Patients, who participated in the study expressed similar needs regardless of age. However, younger cancer patients more frequently had a need for better services from the hospital (Q14; *p* = 0.01), need to have more economic insurance information in relation to their illness (Q15; *p* = 0.01) and economic help (Q16; *p* < 0.001) in comparison to older cancer patients. Whereas older patients more frequently expressed the need to speak with a spiritual advisor (Q18; *p* < 0.001) than those who were younger (Table 3).

**Table 3:** Unmet needs expressed by patients

	Age	Gender			Education				Place of residence			Professional activity			
		female	male		primary	secondary	high		city	village		active	unemployed	pensioner	
Item number	*p*^	*n* /%	*n* /%	p*	*n* /%	*n* /%	*n* /%	p*	*n* /%	*n* /%	p*	*n* /%	*n* /%	*n* /%	p*
Q1	0.61	225 /44	305 /55	< 0.001	89 /49	366 /51	71 /48	0.82	335 /50	196 /51	0.63	114 /48	21 /51	395 /51	0.78
Q2	0.42	338 /67	390 /71	0.35	115 /64	514 /71	92 /63	0.05	461 /68	267 /70	0.67	164 /69	28 /68	534 /68	0.93
Q3	0.16	268 /53	311 /56	0.28	103 /57	400 /55	70 /48	0.13	366 /54	214 /56	0.77	130 /55	24 /59	424 /54	0.93
Q4	0.37	293 /58	342 /62	0.16	105 /58	442 /61	80 /54	0.23	408 /60	227 /59	0.65	147 /62	24 /59	462 /59	0.79
Q5	0.07	217 /43	279 /50	0.02	87 /48	342 /47	61 /41	0.39	297 /44	199 /52	0.03	113 /48	21 /51	361 /46	0.78
Q6	0.70	246 /49	308 /56	0.02	101 /56	382 /53	64 /44	0.06	340 /50	214 /56	0.09	123 /52	22 /54	409 /52	0.92
Q7	0.90	276 /54	331 /60	0.09	114 /63	408 /56	76 /52	0.08	372 /55	235 /61	0.06	125 /53	20 /49	462 /59	0.11
Q8	0.28	212 /42	295 /53	< 0.001	99 /55	341 /47	61 /41	0.05	309 /46	199 /52	0.05	107 /45	16 /39	385 /49	0.20
Q9	0.87	239 /47	305 /55	0.009	106 /59	365 /50	66 /45	0.02	337 /50	207 /54	0.32	118 /50	23 /56	402 /52	0.72
Q10	0.49	52 /10	80 /14	0.04	28 /16	77 /11	26 /18	0.02	78 /12	54 /14	0.25	27 /11	5 /12	100 /13	0.86
Q11	0.22	136 /27	159 /29	0.53	54 /30	195 /27	42 /29	0.68	177 /26	119 /31	0.09	64 /27	9 /22	223 /29	0.57
Q12	0.15	138 /27	172 /31	0.13	65 /36	197 /27	43 /29	0.05	187 /28	124 /32	0.11	61 /26	5 /12	245 /31	0.009
Q13	0.69	278 /55	335 /61	0.05	103 /57	417 /58	84 /57	0.89	383 /57	230 /60	0.24	134 /57	21 /51	458 /59	0.54
Q14	0.01	288 /57	301 /54	0.51	90 /50	402 /56	90 /61	0.20	372 /55	217 /57	0.55	130 /55	25 /61	432 /55	0.89
Q15	0.01	317 /63	326 /59	0.25	111 /62	441 /61	84 /57	0.40	387 /57	255 /67	0.002	147 /62	29 /71	466 /60	0.44
Q16	< 0.001	155 /31	174 /31	0.76	65 /36	225 /31	34 /23	0.02	189 /28	140 /37	0.004	74 /31	24 /59	231 /30	< 0.001
Q17	0.33	133 /26	121 /22	0.08	51 /28	156 /22	40 /27	0.09	146 /22	106 /28	0.03	45 /19	9 /22	198 /25	0.12
Q18	< 0.001	101 /20	115 /21	0.76	50 /28	131 /18	34 /23	0.008	127 /19	89 /23	0.07	36 /15	5 /12	174 /22	0.02
Q19	0.27	197 /39	228 /41	0.38	86 /48	278 /38	57 /39	0.03	262 /39	164 /43	0.22	85 /36	11 /27	329 /42	0.04
Q20	0.77	172 /34	217 /39	0.07	90 /50	255 /35	39 /27	< 0.001	228 /34	160 /42	0.007	84 /36	15 /37	287 /37	0.93
Q21	0.52	252 /50	314 /57	0.03	108 /60	389 /54	62 /42	< 0.001	346 /51	219 /57	0.06	118 /50	22 /54	424 /54	0.39
Q22	0.87	178 /35	186 /34	0.60	79 /44	232 /32	48 /33	0.003	218 /32	145 /38	0.05	80 /34	15 /37	267 /34	0.96
Q23	0.82	168 /33	209 /38	0.09	71 /39	249 /34	55 /37	0.34	229 /34	148 /39	0.14	89 /38	13 /32	275 /35	0.66

	Marital status			Living				Physician in close family or friends		
	married	single		with partner	with someone	alone		no	yes	
Item number	*n* /%	*n* /%	p*	*n* /%	*n* /%	*n* /%	p*	*n* /%	*n* /%	p*
Q1	375 /52	152 /46	0.06	361 /51	75 /45	84 /49	0.37	455 /51	72 /45	0.17
Q2	513 /71	211 /64	0.01	497 /70	111 /67	108 /63	0.17	621 /70	103 /64	0.13
Q3	404 /56	172 /52	0.17	391 /55	91 /55	87 /51	0.45	500 /56	76 /48	0.09
Q4	449 /62	182 /55	0.05	438 /62	89 /54	96 /56	0.19	545 /61	85 /53	0.06
Q5	360 /50	132 /40	0.002	346 /49	75 /45	64 /37	0.03	425 /48	69 /43	0.20
Q6	382 /53	169 /51	0.50	377 /53	84 /51	84 /49	0.56	480 /54	71 /44	0.04
Q7	425 /59	178 /54	0.09	414 /59	95 /57	86 /50	0.12	522 /58	82 /51	0.08
Q8	359 /50	146 /44	0.05	352 /50	74 /45	71 /42	0.06	438 /49	67 /42	0.11
Q9	374 /52	167 /50	0.57	363 /51	84 /51	84 /49	0.81	468 /52	74 /46	0.19
Q10	87 /12	43 /13	0.65	90 /13	14 /8	24 /14	0.22	110 /12	22 /14	0.62
Q11	208 /29	85 /26	0.25	206 /29	43 /26	41 /24	0.31	258 /29	35 /22	0.06
Q12	211 /29	97 /29	0.91	208 /29	50 /30	44 /26	0.55	260 /29	49 /31	0.80
Q13	427 /59	182 /55	0.12	419 /59	90 /54	91 /53	0.12	522 /58	87 /54	0.21
Q14	403 /56	182 /55	0.88	397 /56	88 /53	94 /55	0.86	486 /54	99 /62	0.11
Q15	431 /60	208 /63	0.31	428 /61	104 /63	101 /59	0.63	552 /62	86 /54	0.06
Q16	213 /30	112 /34	0.13	220 /31	47 /28	55 /32	0.80	284 /32	44 /28	0.25
Q17	163 /23	87 /26	0.20	158 /22	41 /25	47 /27	0.32	214 /24	38 /24	0.85
Q18	151 /21	61 /18	0.36	148 /21	31 /19	31 /18	0.67	173 /19	42 /26	0.06
Q19	296 /41	124 /37	0.26	291 /41	64 /39	62 /36	0.49	359 /40	65 /41	0.99
Q20	274 /38	110 /33	0.14	268 /38	63 /38	45 /26	0.02	343 /38	44 /28	0.007
Q21	388 /54	170 /51	0.49	381 /54	91 /55	80 /47	0.18	482 /54	80 /50	0.31
Q22	235 /33	123 /37	0.18	230 /33	58 /35	64 /37	0.48	317 /35	44 /28	0.04
Q23	254 /35	118 /36	0.96	246 /35	64 /39	57 /33	0.57	324 /36	51 /32	0.23

* *p*-value estimated using chi-square test

^ *p*-value estimated using Mann–Whitney test

#### Gender

One third of the assessed needs were indicated as unmet more frequent by men. Men significantly more often than women needed more information about their diagnosis (Q1; respectively: 55% vs 44%; *p* < 0.001), to be more involved in the therapeutic choices (Q5; respectively: 50% vs 43%; *p* = 0.02), clinicians and nurses to give them more comprehensible information (Q6; respectively: 56% vs 49%; *p* = 0.02), to have a better dialogue with clinicians (Q8; respectively: 53% vs 42%; < 0.001). Male cancer patients were more likely than women to have the unmet need for symptoms (pain, nausea, insomnia, etc.) to be better controlled (Q9; respectively: 55% vs 47%; *p* = 0.009) and the need for more help with eating, dressing and going to the bathroom (Q10; respectively: 14% vs 10%; *p* = 0.04). Additionally, men more frequently needed to feel more useful within their family (Q21; respectively: 57% vs 50%; *p* = 0.03) (Table 3).

#### Education

Patients who graduated only from primary school more frequent reported an unmet need for better controlled symptoms of disease in comparison with patients who graduated from secondary school and high school (Q9; respectively: 59% vs 50% vs 45%; *p* = 0.02). The unmet need of help with eating, dressing and going to the bathroom was reported more often among patients who graduated from primary or high school than secondary school (Q10; respectively: 16% vs 18% vs 11%; *p* = 0.02). Patients with primary school as their highest level of formal education significantly more often than patients with secondary or high school education needed economic help (Q16; respectively: 36% vs 31% vs 23%; *p* = 0.02), to be more reassured by their relatives (Q20; respectively: 50% vs 35% vs 27%; *p* < 0.001), to feel more useful within their family (Q21; respectively: 60% vs 54% vs 42%; *p* < 0.001), to feel less abandoned (Q22; respectively: 44% vs 32% vs 33%; *p* = 0.003). Primary school patients were more likely than secondary or high school patients to express the need to speak with a spiritual advisor (Q18; respectively: 28% vs 18% vs 23%; *p* = 0.008) and with people who have had the same experience (Q19; respectively: 48% vs 38% vs 39%; *p* = 0.03) (Table 3).

#### Place of residence

Patients living in a village more frequently than patients living in cities indicated the need to be more involved in the therapeutic choices (Q5; respectively: 52% vs 44%; *p* = 0.03), need to have more economic insurance information in relation to their illness (Q15; respectively: 67% vs 57%; *p* = 0.002), need economic help (Q16; respectively: 37% vs 28%; *p* = 0.004), need to speak with a psychologist (Q17; respectively: 28% vs 22%; *p* = 0.03), need to be more reassured by their relatives (Q20; respectively: 42% vs 34%; *p* = 0.007) (Table 3).

#### Professional activity

There were no significant differences between informative or relational needs associated with professional activity. Pensioners more often expressed the unmet need for better attention from nurses in comparison with patients who were professionally active or unemployed (Q12; respectively: 31% vs 26% vs 12%; *p* = 0.009). Pensioners were also more likely than professionally active respondents and those who were unemployed to report the unmet need to speak with a spiritual advisor (Q18; respectively: 22% vs 15% vs 12%; *p* = 0.02) and to speak with people who have had the same experience (Q19; respectively: 42% vs 36% vs 27%; *p* = 0.04). Unemployed participants more often had the unmet need for economic help than professionally active patients and pensioners (Q16; respectively: 59% vs 31% vs 30%; *p* < 0.001) (Table 3).

#### Marital status

In general patients’ marital status did not correlate with different expressions of unmet needs. In the case of two items there were significant differences. Patients who were married/in a stable relationship were more likely than those who were single to report the unmet needs for more information about their future condition (Q2; respectively: 71% vs 64%; *p* = 0.01) and for being more involved in the therapeutic choices (Q5; respectively: 50% vs 40%; *p* = 0.002) (Table 3).

#### Household

Patients living with a partner or children/another family member were, in comparison with patients living alone, more likely to need to be reassured by their relatives (Q20; respectively: 38% vs 38% vs 26%; *p* = 0.02) and to be involved in therapeutic choices (Q5; respectively: 49% vs 45% vs 37%; *p* = 0.03) (Table 3).

#### Physician as a close family member/friend

Patients who did not have a physician as a close family member or friend more frequently expressed the need for clinicians and nurses to give them more comprehensible information (Q6; respectively: 54% vs 44%; *p* = 0.04), the need to be more reassured by their relatives (Q20; respectively: 38% vs 28%; *p* = 0.007), and the need to feel less abandoned (Q22; respectively: 35% vs 28%; *p* = 0.04) than those who did (Table 3).

#### Multivariate analysis

In multivariate analysis gender (male vs. female), odds ratio [OR]:1.48 (95% confidence interval [CI]:1.15–1.89), *p* = 0.002 significantly increased the need for more information about diagnosis (Q1). Level of education (low vs. high), OR:1.51 (95% CI:1.04–2.19), *p* = 0.03 and marital status (married vs. single), OR:1.38 (95% CI:1.04–1.83), *p* = 0.03 significantly increased the need for more information about future condition (Q2). Age, OR:0.99 (95% CI:0.98–1.00), *p* = 0.04 had significant impact on expressing the need to be more involved in the therapeutic choices (Q5). Gender (male vs. female), OR:1.51 (95% CI:1.16–1.95), *p* = 0.002 and level of education (low vs. high), OR:1.65 (95% CI:1.03–2.65), *p* = 0.04 significantly increased the need for better dialogue with clinicians (Q8). Gender (male vs. female), OR:1.37 (95% CI:1.07–1.76), *p* = 0.01 and level of education (low vs. high), OR:1.70 (95% CI:1.09–2.67), *p* = 0.02 significantly increased the need for better control of symptoms (Q9). Gender (male vs. female), OR:1.60 (95% CI:1.10–2.35), *p* = 0.02 and level of education (low vs. high), OR:0.49 (95% CI:0.30–0.80), *p* = 0.005 had significant impact on expressing the need for help in daily activities (Q10). Professional activity (unemployed vs. active), OR:0.35 (95% CI:0.13–0.95), *p* = 0.04 had significant impact on expressing the need for better attention from nurses (Q12). Age, OR:0.98 (95% CI:0.97–0.99), *p* = 0.02 and place of residence (village vs. city), OR:1.48 (95% CI:1.13–1.93), *p* = 0.005 had significant impact on expressing the need for more economic insurance information (Q15). Age, OR:0.97 (95% CI:0.96–0.99), *p* = 0.002; level of education (low vs. high), OR:1.99 (95% CI:1.17–3.39), *p* = 0.01; place of residence (village vs. city), OR:1.36 (95% CI:1.02–1.80), *p* = 0.03 and professional activity (unemployed vs. active), OR:2.52 (95% CI:1.26–5.06), *p* = 0.009 had significant impact on expressing the need for economic help (Q16). Place of residence (village vs. city), OR:1.42 (95% CI:1.05–1.92), *p* = 0.02 significantly increased the need to speak with a psychologist (Q17). Have a doctor as a close family member/friend (no vs. yes), OR:0.64 (95% CI:0.43–0.97), *p* = 0.03 had significant impact on expressing the need to speak with a spiritual advisor (Q18). Level of education (low vs. high), OR:3.02 (95% CI:1.81–5.03), *p* < 0.001 and household (living alone vs. living with partner), OR:0.57 (95% CI:0.38–0.84), *p* = 0.004 had significant impact on expressing the need to be more reassured by relatives (Q20). Level of education (low vs. high), OR:2.14 (95% CI:1.34–3.40), *p* = 0.001 significantly increased the need to feel more useful within family (Q21).

### Qualitative analysis

An extra interview and VPQ were conducted on 71 patients (Table 4).

**Table 4:** Study subgroup demographics

	*n*	%
Gender		
female	49	69.0
male	22	31.0
Age: range 30–88 years, median 66 (50–70) years (25–75% IQR)		
Education		
primary	6	8.5
secondary	39	54.9
high	26	36.6
Place of residence		
city	54	76.1
village	17	23.9
Professional activity		
active	25	35.2
unemployed	1	1.4
pensioner	45	63.4
Marital status		
married	51	71.8
relationship broken down during disease or in relation to disease	0	0.0
single	20	28.2
Living with		
partner	31	43.7
child/children and/or another family member	27	38.0
alone	13	18.3
Doctor in close family or friends		
yes	11	15.5
no	60	84.5
Cancer		
head and neck	6	8.5
upper digestive system	0	0.0
lower digestive system	10	14.0
lung	7	9.9
breast	26	36.6
gynecological	6	8.5
prostate	11	15.5
brain	2	2.8
urinary system	0	0.0
no data	3	4.2
Time from cancer diagnosis: range 1–68 months, median 4 (2–6) months (25–75% IQR)		

IQR – interquartile range

#### Understanding of the meaning of the unmet non-medical needs

Eleven items with the prevalence of need of at least 50% were analyzed. The NEQ questions are relatively simple. On the other hand, each need may be perceived differently by individual patients. In fact, the qualitative analysis showed the multiplicity of understanding (Table 5).

**Table 5:** Meanings of unmet need in patients’ perspectives

Q1	I need more information about diagnosis
	Patients wished to know:
	the etiology of the disease
	how the disease originated
	how the disease will progress
	how the disease will go on
	the prognosis
	the possible treatment
	how the treatment will proceed
	what the abbreviations mean (for example HER)
Q2	I need more information about my future condition
	Patients wished to know:
	what they can expect in the future
	what's ahead of them
Q3	I need more information about the exams I am undergoing
	Patients wished to know:
	what kind of exams will be done
	in which sequence exams will be done
	why those exams should be done
	what results can they expect from those exams
Q4	I need more explanations of treatments
	Patients wished to know:
	why a particular treatment was selected
	what outcomes of treatment are expected
	are there any clinical trials suitable for them
Q6	I need clinicians and nurses to give me more comprehensible information
	Patients wished:
	to get information with clearer vocabulary
Q7	I need clinicians to be more sincere with me
	Patients wished physicians:
	to be more sincere
	to tell the truth
	to be not afraid that bad news will negatively affect to them and make them sad
Q9	I need my symptoms (pain, nausea, insomnia, etc.) to be better controlled
	Patients wished:
	to reduce pain
	physicians to pay more attention to their symptoms
Q13	I need to be more reassured by the clinicians
	Patients wished:
	physicians to give hope
	to get extra options for treatment
Q14	I need better services from the hospital (bathrooms, meals, cleaning)
	Patients wished:
	more tasty meals
	to have the possibility to choose meals
	diet more appropriate to their health condition
Q15	I need to have more economic insurance information (tickets, invalidity, etc.) in relation to my illness
	Patients wished:
	to get more appropriate information
	to be more clearly informed
	to get written information
Q21	I need to feel more useful within my family
	Patients wished:
	their families not to feel sorry for them
	to be useful, helpful
	not to feel useless

#### Evaluation of the questionnaire

The patients’ subjective evaluation of the survey was assessed during the interview with a psychologist. Patients considered that the NEQ is a good instrument to express their needs. Overall they were glad that someone was interested in their needs and discussing their problems. For 87% of patients, the questions were generally understandable, and 83% of them had no difficulty answering the questions. 80% of patients considered that completing the questionnaire may facilitate better contact with a physician/nurse/other professionals. In patients’ opinions, the results of the study using this questionnaire could improve patient-doctor relations.

Patients who completed the NEQ, declared that they are now more aware of their non-medical needs. They considered that developing informational brochures to help address the unmet needs included in the NEQ would be helpful.

## Discussion

A key component of both high quality care for cancer patients and the satisfaction of patients and their families seems to be the complete assessment of patient needs. There are different areas of patients’ non-medical needs: needs related to information and communication with clinicians [10, 19–24], psycho-social support [19, 21, 23, 25], relationships [10], assistance/care [22, 26], economic support and insurance information [21, 22], spiritual issues [27] and sexual issues [28, 29].

Some needs of cancer patients seem to be unmet – a patient could experience a gap between what they need and what they actually receive. In a previous study [30] 384 Polish cancer patients were analyzed and it was discovered that only 21% of them received professional support from a psychologist and 4% of patients from a priest. In addition, only 7% of participants received help from social worker. Yet it is known that psychological, spiritual and social support has an important role in patients’ well-being during cancer care.

Most cancer patients reported at least one unmet need [31–33]. In the current study, only 6% of patients did not indicate any of 23 analyzed items. Eleven of 23 items were highlighted as unmet needs by at least 50% of participants. The unmet needs were mainly informative needs about the diagnosis, exams, treatment, prognosis, and about economic insurance. In a previous pilot study conducted among 160 male lung cancer patients, informative needs were the most frequently expressed [18]. More than half of Italian cancer patients expressed needs for more information about diagnosis, future conditions, exams and treatment [12, 13]. Geraldine et al. [34] carried out depth interview study of informative needs among English cancer patients and determined that patients wanted information about diagnosis, therapeutic options, and side effects of treatment. In a systemic review of cancer patients’ needs conducted by Webb et al. [5] almost all studies showed needs for information and these needs were the most commonly expressed. A systematic review of 30 studies of unmet needs in newly diagnosed older cancer patients [33] also noted that the most common needs were informative needs.

Bonacchi et al. [15] determined that informative unmet needs were changing significantly over time: informative needs for more information about diagnosis, future conditions or treatment were mostly expressed at cancer diagnosis and also at confirmation of progression [15]. Similarly, Hsieh et al. [35] found that lung cancer patients expressed the highest rate of information needs at the time of diagnosis. During the examinations and initial treatment, patients would often like to receive information to better understand and cope with cancer [35, 36]. Later, patients predominantly had information needs regarding the prognosis and how to identify possible cancer recurrence [37]. A systematic review of studies regarding unmet needs of older cancer patients [33] found that the level of unmet needs was the highest after diagnosis and at the beginning of the treatment, decreasing over time.

Cancer patients wanted to be well-informed about diagnosis, treatment options, possible side effects and prognosis, and most of them expressed the need for as much information as possible from their doctors, whether the update was bad or good [38]. Information about possible side effects, treatment, confirmation of cancer, likelihood of cure, whether all parts of the body are involved, the way treatment works and day-to-day progression were perceived as absolutely necessary by at least half of patients analyzed [24]. Mokhles et al. [39] demonstrated that most lung cancer patients received only general information and only 20% of them received information about prognosis. Some patients indicated that doctors do not always give them sufficient or understandable information [40, 41]. It is well known that the information provided for patients could have a beneficial impact on their feelings, reduce anxiety and allow them plan for the future [42–44]. Meeting the need for information, even if the new is bad, increases hope [24]. Adequate understanding of the disease could influence thoughts about the disease, the approach to treatment and improve quality of life [45–49]. Additionally, a correlation between unmet needs and distress was presented [12, 15]. Distressed patients had higher levels of unmet needs across all areas investigated [12]. A higher resilience has helped patients find satisfaction with care and staff and get their needs more comprehensively met [12].

Polish cancer patients seem to be dissatisfied with the patient-doctor communication [50, 51]. Physicians are often not familiar with standards for how to provide cancer patients with information about diagnosis, prognosis, etc. [50, 52, 53]. Although patients wanted simple, clear answers to their questions, Polish patients’ informative needs seem not to be met. In the current study patients indicated the need for more information about diagnosis (50%), exams (54.6%) and treatments (59.9%). In an Italian study using the same questionnaire only 30–40% of patients needed the information highlighted as required by this study [10, 13]. Only the informative need for future condition was similar among Polish and Italian cancer patients (respectively: 68.7% and 61/68%) [10, 13].

Clinical and demographic factors have an influence on the perception of disease and non-medical needs. The results from this study showed that subgroups of patients experience different needs. For example, although patients are seeking more information in general, individuals who have a physician in close family or friends expressed significantly less informative needs than those who do not. In the current study gender was associated with unmet needs. Men significantly more often than women needed additional information. Moreover, men significantly more frequent than women expressed a need for their symptoms to be better controlled and for support in daily life activities. Additionally men in particular would like to feel more useful within their family. In some other studies, the gender association was the opposite. Hsieh et al. [35] determined that female lung cancer patients had higher informative needs related to treatment than men. Bonacchi et al. [12] determined that women more frequently than men had material needs and needs for psycho-emotional support [12].

In this study patients, who graduated from primary school significantly more often than patients with secondary and high school education needed more economic help and psychological support (from their relatives, spiritual advisor or patients with the same experience). In an Italian study [12] using the NEQ, primary school patients more often needed support in daily life activities or better attention from nurses than patients with secondary and higher education. Bonacchi et al. [12] showed that high number of unmet needs was associated with a lower level of formal education. Lung cancer patients with a higher education level were more likely to express the informative needs than less educated participants [35]. In this study those associations were not observed.

Patients who live in a village were more likely to express the need to have more economic insurance information and more psychological support than those who live in cities. While pensioners in comparison with patients who were professionally active or unemployed would more frequently like to speak with a spiritual advisor and with people who have had the same experience. Pensioners more frequently than those patients who were professionally active expressed unmet needs for better attention from nurses. Unemployed participants more frequent had an unmet need for economic help than professionally active patients or pensioners.

Bonacchi et al. [12] did not confirm a relationship between informative needs and marital status, but single people were more likely to have needs for psycho-emotional support than those who were married. In the study conducted among lung cancer patients, participants who had children were more likely than those with no children to need information related to the disease [35]. In the current study married patients wanted to receive more information about their future condition more often than those who were single. Patients who did not have a doctor in close family or friends were more likely to express the need to be more reassured by their relatives.

Wang et al. [54] noted that qualitative study enables the identification of needs that would not have been recognized in a quantitative analysis. Additionally, patients can detail their unmet needs, therefore these needs could be more precisely identified. For better understanding the unmet needs of Polish cancer patients a qualitative analysis was provided in the current study. Individual items of the NEQ were defined in a simple way. This analysis showed that patients understood the items of this questionnaire. However, the meaning of individual needs as interpreted by patients is broad and not homogeneous. Therefore, patients' perceptions of a specific need may not be the same. Tamburini et al. [10] carried out a quantitative and qualitative analysis among Italian cancer patients using the same instrument as employed in current study. The most frequent indicated need, as in this study, was “I need more information about my future condition”. Both patients from Italy and Poland specifically wanted to know the probability of being cured, how their future life will be, how they will feel and what they will be able to do. Instead of generalized statistical prognoses, they wished to receive more personalized explanations based on an individual approach to each patient.

The current study showed wide range of non-medical needs among Polish cancer patients. These needs in general are not adequately met. The reason of that could be caused by medical staff priorities – cancer cure. The modern cancer care goes through the holistic approach. From patients’ site their well-being is not only good physical condition but also psychosocial and spiritual fulfilment. To ensure good quality of life of cancer patients, the recognizing the unmet needs is essential. Therefore some instruments to assess cancer patients’ needs are introduced [2]. The NEQ seems to be a good tool, also as it was showed in current study among Polish population. Using the NEQ in daily clinical practice could be very useful to identify non-medical needs of a particular cancer patient and could supplement the scope of medical care. Introduction of an instrument to estimate unmet needs could be a challenge in better, holistic and personalized cancer patients’ care. Unfortunately, so far there are no recommendations created by science societies or governments for identification of the non-medical needs of cancer patients.

### Limitations

This study has some limitations. Participants were recruited from seven cancer centers in Poland, but numbers of individuals of each centers were different. Patients in qualitative analysis came from only two centers. The study did not analyze the differences in expression of unmet needs due to cancer type, time from diagnosis and intention of treatment. There were no measurements of mental status, anxiety and depression.

## Conclusion

Most Polish cancer patients have some unmet non-medical needs, especially informative needs, material needs and needs for a psychoemotional support. More than half of patients (50–69%) expressed a lack of information: needs for more information about diagnosis, examinations and treatments, needs for information about their future condition. Most of participants (61%) expressed the need to have more information about economic insurance in relation to their illness. Therefore, the more attention should be paid for good communication between medical staff and patient and giving more sufficient information about diagnosis, treatment, future condition, etc. Unmet needs were more frequent among men, with lower level of formal education, those living in village and pensioners. This sociodemographic group of cancer patients requires a special assistance.

### Supplementary Information

Below is the link to the electronic supplementary material.

Supplementary file1 (ZIP 67.5 KB)

## Data Availability

The datasets used and/or analyzed during the current study are available from the corresponding author upon reasonable request.
