# Supplementary material for: Unmet non-medical needs of cancer patients in Poland: a quantitative and qualitative study
Source: Support Care Cancer. 2024 Feb 22;32(3):183. doi: 10.1007/s00520-024-08387-5 (PMC10884169; doi:10.1007/s00520-024-08387-5)
Supplement: Supplementary file 1 — Supplementary file1 (ZIP 67.5 KB) [file 520_2024_8387_MOESM1_ESM.zip › Supplementary material/NEQ English version.docx]

QUESTIONNAIRE NUMBER……………………..

1. I need more information about my diagnosis

- Yes
- No

1. I need more information about my future condition

- Yes
- No

1. I need more information about the exams I am undergoing

- Yes
- No

1. I need more explanations of treatments

- Yes
- No

1. I need to be more involved in the therapeutic choices

- Yes
- No

1. I need clinicians and nurses to give me more comprehensible information

- Yes
- No

1. I need clinicians to be more sincere with me

- Yes
- No

1. I need to have a better dialogue with Clinicians

- Yes
- No

1. I need my symptoms (pain, nausea, insomnia, etc.) to be better controlled

- Yes
- No

1. I need more help with eating, dressing, and going to the bathroom

- Yes
- No

1. I need better respect for my intimacy

- Yes
- No

1. I need better attention from nurses

- Yes
- No

1. I need to be more reassured by the clinicians

- Yes
- No

1. I need better services from the hospital (bathrooms, meals, cleaning)

- Yes
- No

1. I need to have more economic insurance information (tickets, invalidity, etc..) In relation to my illness

- Yes
- No

1. I need economic help

- Yes
- No

1. I need to speak with a psychologist

- Yes
- No

1. I need to speak with a spiritual advisor

- Yes
- No

1. I need to speak with people who have this same experience

- Yes
- No

1. I need to be more reassured by my relatives

- Yes
- No

1. I need to feel more useful within my family

- Yes
- No

1. I need to feel less abandoned

- Yes
- No

1. I need to receive less commiseration from other people

- Yes
- No

DEMOGRAPHIC AND CLINICAL DATA

1. Gender:

- female
- male

1. Age: …………years
2. Education:

- primary
- secondary
- high

1. Place of residence

- city, ….…………….
- village

1. Professional activity

- student
- active
- active, but actually sick leave
- unemployed
- pensioner
- disability pensioner

1. Marital status

- married or in a stable informal relationship
- relationship broken during disease or in relation to disease
- single
- divorced
- widow/widower

1. Living with:

- partner
- partner and child/children
- child/children
- another family member
- alone

1. Do you have a medical doctor as a close family or friend?

- yes
- no

1. Cancer:………………………………………………………..
2. Approximate date of cancer diagnosis:…………………………

(month/year)
