# Supplementary material for: Unmet non-medical needs of cancer patients in Poland: a quantitative and qualitative study
Source: Support Care Cancer. 2024 Feb 22;32(3):183. doi: 10.1007/s00520-024-08387-5 (PMC10884169; doi:10.1007/s00520-024-08387-5)
Supplement: Supplementary file 1 — Supplementary file1 (ZIP 67.5 KB) [file 520_2024_8387_MOESM1_ESM.zip › Supplementary material/NEQ Polish version.docx]

KWESTIONARIUSZ Nr ……..

1. Potrzebuję więcej informacji na temat mojej choroby

- Tak
- Nie

1. Potrzebuję więcej informacji na temat mojego stanu zdrowia w przyszłości

- Tak
- Nie

1. Potrzebuję więcej informacji dotyczących wykonywanych badań

- Tak
- Nie

1. Potrzebuję więcej informacji na temat mojego leczenia

- Tak
- Nie

1. Chciałabym/chciałbym w większym stopniu uczestniczyć w podejmowaniu decyzji dotyczących mojego leczenia

- Tak
- Nie

1. Chciałabym/chciałbym, aby lekarze i pielęgniarki przekazywali mi informacje bardziej zrozumiałym językiem/w bardziej zrozumiały sposób

- Tak
- Nie

1. Chciałabym/chciałbym, aby lekarze byli ze mną bardziej szczerzy

- Tak
- Nie

1. Potrzebuję bliższego kontaktu z lekarzem

- Tak
- Nie

1. Chciałabym/chciałbym, aby moje objawy (np. ból, nudności, bezsenność) były lepiej łagodzone

- Tak
- Nie

1. Potrzebuję więcej pomocy przy czynnościach takich jak jedzenie, ubieranie się, korzystanie z toalety

- Tak
- Nie

1. Potrzebuję większego poszanowania mojej intymności/prywatności

- Tak
- Nie

1. Chciałabym/chciałbym, aby pielęgniarki okazywały mi więcej uwagi/zainteresowania

- Tak
- Nie

1. Chciałabym/chciałbym, aby lekarze umieli rozpraszać moje wątpliwości

- Tak
- Nie

1. Chciałabym/chciałbym, aby w szpitalu były lepsze warunki (np. stan łazienek, jakość posiłków)

- Tak
- Nie

1. Potrzebuję więcej informacji dotyczących należnych mi świadczeń socjalnych związanych z moją chorobą

- Tak
- Nie

1. Potrzebuję pomocy finansowej

- Tak
- Nie

1. Chciałabym/chciałbym mieć możliwość spotkania/rozmowy z psychologiem

- Tak
- Nie

1. Chciałabym/chciałbym mieć możliwość spotkania/rozmowy z księdzem

- Tak
- Nie

1. Chciałabym/chciałbym mieć możliwość spotkania/rozmowy z osobami, które mają taką samą chorobę

- Tak
- Nie

1. Chciałabym/chciałbym, aby moi bliscy bardziej mnie wspierali

- Tak
- Nie

1. Chciałabym/chciałbym czuć się bardziej potrzebna/y mojej rodzinie

- Tak
- Nie

1. Chciałabym/chciałbym czuć się mniej samotna/y

- Tak
- Nie

1. Chciałabym/chciałbym, aby ludzie okazywali mi mniej współczucia/litości

- Tak
- Nie

METRYCZKA Nr ………

1. Płeć:

- kobieta
- mężczyzna

1. Wiek: …………lat
2. Wykształcenie:

- podstawowe
- średnie/zawodowe
- wyższe

1. Miejsce zamieszkania

- miasto, jakie ….…………….
- wieś

1. Aktywność zawodowa

- uczeń/student
- nadal pracuję zawodowo
- w chwili obecnej L4 lub zasiłek rehabilitacyjny
- bezrobotny
- emeryt
- rencista

1. Stan cywilny

- mężatka/żonaty lub w stałym związku
- mój związek zakończył się w trakcie lub w związku z chorobą
- panna/kawaler
- rozwiedziona/rozwiedziony
- wdowa/wdowiec

1. Mieszkam

- tylko ze współmałżonkiem lub partnerem/partnerką
- ze współmałżonkiem lub partnerem/partnerką i z dzieckiem/dziećmi
- tylko z dzieckiem/dziećmi
- z innym członkiem rodziny
- samotnie

1. Czy ktoś z Pani/Pana najbliższej rodziny lub przyjaciół jest lekarzem?

- tak
- nie

1. Rozpoznanie choroby:……………………………………………
2. Przybliżona data rozpoznania choroby:…………………………

(miesiąc/rok)
