# Supplementary material for: Unmet non-medical needs of cancer patients in Poland: a quantitative and qualitative study
Source: Support Care Cancer. 2024 Feb 22;32(3):183. doi: 10.1007/s00520-024-08387-5 (PMC10884169; doi:10.1007/s00520-024-08387-5)
Supplement: Supplementary file 1 — Supplementary file1 (ZIP 67.5 KB) [file 520_2024_8387_MOESM1_ESM.zip › Supplementary material/VPQ English version.docx]

VALIDATION No ……….

1. **Time of completion the questionnaire**
2. main questionnaire: ……….. minutes
3. demographics: ….……….. minutes
4. **Comprehensibility and acceptability of the questionnaire**
5. Is the form of the questionnaire good in your opinion?

- Yes
- No

1. Is the font size big enough in your opinion?

- Yes
- No

1. Do you think that the questionnaire is sufficiently long?

- Yes
- No – should be shorter
- No – should be longer

1. Are the questions generally understandable in your opinion?

- Yes
- No

1. Are any questions difficult for you to answer clearly?

- Yes

Which? Number of question ………..

- No

1. Are any questions you do not want to answer?

- Yes

Which? Number of question ………..

- No

1. Is there anything else you would like to tell about your needs?

- Yes

What?………………………………………………………………………...

- No

1. Do you think that completing this questionnaire may facilitate better contact with the doctor / nurse / other staff?

- Yes
- No

1. Did you identify any important needs, which you did not recognize before the questionnaire?

- Yes

What?...........................................................................................................

Number of question ………..

- No
