# Supplementary material for: Unmet non-medical needs of cancer patients in Poland: a quantitative and qualitative study
Source: Support Care Cancer. 2024 Feb 22;32(3):183. doi: 10.1007/s00520-024-08387-5 (PMC10884169; doi:10.1007/s00520-024-08387-5)
Supplement: Supplementary file 1 — Supplementary file1 (ZIP 67.5 KB) [file 520_2024_8387_MOESM1_ESM.zip › Supplementary material/VPQ Polish version.docx]

WALIDACJA Nr ……….

1. **Czas wypełniania ankiety**
2. właściwej ankiety: ……….. minut
3. metryczki: ….…………….. minut
4. **Zrozumiałość, akceptowalność ankiety**
5. Czy forma ankiety jest według Pani/Pana dobra?

- Tak
- Nie

1. Czy wielkość liter jest według Pani/Pana wystarczająco duża?

- Tak
- Nie

1. Czy sądzi Pani/Pan, że ankieta jest odpowiednio długa?

- Tak
- Nie – powinna być krótsza
- Nie – powinna być dłuższa

1. Czy pytania są według Pani/Pana generalnie zrozumiałe?

- Tak
- Nie

1. Czy były pytania, na które trudno było Pani/Panu odpowiedzieć jednoznacznie?

- Tak

Które? Nr Pytania………..

- Nie

1. Czy były pytania, na które Pani/Pan nie mieli ochoty odpowiadać?

- Tak

Które? Nr Pytania………..

- Nie

1. Czy jest coś jeszcze, o czym Pani/Pan chciałby powiedzieć odnośnie swoich potrzeb?

- Tak

Co takiego?………………………………………………………………………...

- Nie

1. Czy myśli/sądzi Pani/Pan, że wypełnienie takiej ankiety może pomóc w lepszym kontakcie z lekarzem/pielęgniarką/innym personelem?

- Tak
- Nie

1. Czy dzięki tej ankiecie zauważyła Pani/Pan coś ważnego, na co wcześniej nie zwracała Pani/Pan uwagi?

- Tak

Co takiego?...........................................................................................................

Ewentualnie Nr Pytania ………..

- Nie
