# Supplementary material for: Unmet non-medical needs of cancer patients in Poland: a quantitative and qualitative study
Source: Support Care Cancer. 2024 Feb 22;32(3):183. doi: 10.1007/s00520-024-08387-5 (PMC10884169; doi:10.1007/s00520-024-08387-5)
Supplement: Supplementary file 1 — Supplementary file1 (ZIP 67.5 KB) [file 520_2024_8387_MOESM1_ESM.zip › Supplementary material/consent form.doc]

# ZGODA NA UCZSTNICTWO W BADANIU

Świadomie i dobrowolnie wyrażam zgodę na uczestnictwo w badaniu pt.: „Walidacja standaryzowanego kwestionariusza ankietowego NEQ (Needs Evaluation Questionnaire) w polskiej populacji pacjentów z chorobą nowotworową, analiza jakościowa i ilościowa potrzeb pozamedycznych pacjentów”.

Wyrażam zgodę na wykorzystanie uzyskanych informacji do celów naukowych i ewentualnej publikacji.

## Udział w badaniu jest anonimowy. Żadne dane osobowe nie zostaną ujawnione. Ochrona danych osobowych zgodnie z Ustawą z dnia 10 maja 2018 r. o ochronie danych osobowych.

............................................... ..........................................................

Data	Podpis badanego

............................................... ..........................................................

Data	Podpis osoby informującej
